# Subterranean Clover and Sulla as Valuable and Complementary Sources of Bioactive Compounds for Rainfed Mediterranean Farming Systems

**DOI:** 10.3390/plants12020417

**Published:** 2023-01-16

**Authors:** Maria Giovanna Molinu, Leonardo Sulas, Giuseppe Campesi, Giovanni Antonio Re, Federico Sanna, Giovanna Piluzza

**Affiliations:** 1 National Research Council, Institute of Sciences of Food Production, Traversa La Crucca 3, Località Baldinca, 07100 Sassari, Italy; 2 National Research Council, Institute for the Animal Production System in Mediterranean Environment, Traversa La Crucca 3, Località Baldinca, 07100 Sassari, Italy

**Keywords:** *Sulla coronaria*, Trifolium subterraneum, fodder, growth stage, phenolic compounds

## Abstract

Mediterranean pasture and forage legumes are important components of sustainable production systems. Subterranean clover and sulla represent key species having proven high agronomic value and traits for production and multiple services. Our research investigated the potential of the abovementioned species as a source of phenolic compounds and antioxidants for contributing to support their full exploitation in the fodder, animal welfare, and nutraceutical sectors. Antioxidant capacity, as well as the content of total phenolic compounds and individual phenolic compounds, was determined in subterranean clover and sulla shoots at the vegetative, flower bud, flowering, and seed ripening phenological stages. The antioxidant capacity and the phenolic content were affected significantly by harvest time. In subterranean clover, 10 individual phenolic compounds were detected, and isoflavones were the most abundant (3.19–18.27 mg·g^−1^ DM). Eleven phenolic compounds were identified in sulla shoots, and chlorogenic acid (0.76–3.43 mg·g^−1^ DM) and diosmin (3.64–4.94 mg·g^−1^ DM) were the most represented compounds at the vegetative and flower bud stage. On the basis of our findings, a complementary utilization of both legume species is suggested; this might ensure the exploitation of all phenolic compounds in view of the potential benefits for animal production and health.

## 1. Introduction

In Mediterranean areas, pasture and forage legumes represent key components of sustainable production systems supplying high-quality fodder and fixing relevant amounts of atmospheric nitrogen [1,2]. They also allow for a wide range of alternative uses and important multiple services within diversified production systems from agro-silvopastoral contexts to organic and conventional agriculture [3].

To cope with the very high environmental variability, Mediterranean pasture and forage legumes have developed distinctive strategies to ensure adaptation and reproduction [4,5]. The key requirements for the persistence of annual self-reseeding legumes are (i) that an adequate number of seeds must be produced and must survive under grazing and (ii) that breakdown of hardseededness should ensure adequate seedling number at the onset of the following productive seasons [5]. Otherwise, the persistence of perennial legumes is typically based on the vegetative regeneration ability of mother plants after summer that is coupled with drought tolerance traits. This paper focuses on two flagship legume species for rainfed Mediterranean areas, one annual and one perennial, which can be concurrently found as native plants and/or as cultivated stands according to different environments and/or contexts of utilization [2,6].

*Trifolium subterraneum* L. (subterranean clover) *sensu lato* [7], whose center of origin is the Mediterranean basin and West Asia, is one of the most important annual self-reseeding pasture legumes, which has been widely studied with the main aim of selecting commercial varieties in Australia [8] and in Italy [9]. Subterranean clover proved to be the most widespread spontaneous pasture legume species in Sardinia [10,11,12], as well as an invaluable worldwide stock of germplasm [13]. On the basis of germplasm collected in the Mediterranean basin, more than 50 cultivars have been released since 1900 in Australia. From its initial accidental introduction into Australia in the 19th Century, subterranean clover has become the most widely sown annual pasture legume species in southern Australia (over 29 million ha), valued in the livestock and grain industries as a source of high-quality forage and for its ability to fix atmospheric nitrogen [14,15]. Several studies have documented its valuable traits in different parts of the world and contexts of utilization [8,9]. The genetic improvement of subterranean clover and its development as an important annual pasture legume, as well as a source of germplasm, in addition to cultivar development and the traits exploited to date, were reviewed by Nichols et al. [15,16]. Subterranean clover produces specific secondary metabolites, biologically active, belonging to a subclass of flavonoids, called isoflavones, namely, formononetin, genistein, and biochanin. Their chemical structure contains a C15 skeleton composed of an aromatic ring, condensed with a heterocyclic ring, and a third aromatic ring connected to these in position 3 instead of 2, as in the more common flavonoids. Isoflavones are phytoalexins, low-molecular-weight compounds synthesized and accumulated in plants in response to biotic and abiotic stress. Additionally, they are also synthesized thanks to the symbiotic relationship established with rhizobial bacteria [17]. 

These compounds have fungistatic, antibacterial, antiviral, and antioxidant properties; their concentration rises during stress and is influenced by environmental and climatic conditions, as well as by harvest time [18]. Isoflavones may exhibit a variety of biological functions such as antiaging and anticancer, and they may protect against cardiovascular diseases and microbiome modulations [18]. Isoflavones are also called phytoestrogens due to their similar chemical structural to 17-β-estradiol; in fact, they can bind to both estrogen ERα and ERβ receptors, thus exhibiting both estrogenic and antiestrogenic properties [18]. These compounds being estrogenically active were found to be responsible for ewe infertility in Australia [19,20]. Therefore, subterranean clover breeding programs have aimed to select genotypes with low content of the estrogenic isoflavone formononetin [15,21]. It is worth noting that a low formononetin content, such as that present in the newly released varieties of subterranean clover, did not appear to interfere negatively with the reproductive processes of ewes and seemed to favor body growth [22].

*Sulla coronaria* (L.) Medik (sulla) syn. *Hedysarum coronarium* L. [23] is a short-lived perennial legume native to the central–western Mediterranean basin with remarkable adaptation to marginal and drought-prone environments. Usually, it is grown in Mediterranean countries for hay and silage, or as a pasture plant or as a rainfed biennial forage crop [24,25,26]. Sulla has noticeable potential in fixing atmospheric nitrogen and improving soil fertility and structure, as well as a valuable role in supporting multifunctional agriculture [27]. It is worth noting that the Italian sulla germplasm, due to its large genetic diversity and variability, is a reservoir of variation for important agronomic traits such as wide adaptation across both drought-prone and cold-prone environments [28,29]. Sulla produces a bloat-safe and high-quality forage with a proper content of condensed tannins, which is beneficial for improving animal health and welfare [30,31]. 

Procedures for the quantification of tannins in leguminous forage plants, and their beneficial effects and role in animal husbandry and environmental sustainability were reviewed by Piluzza et al. [31]. Additionally, plant bioactive compounds are being widely studied as natural phytochemicals due to their greater antioxidant properties, compared with synthetic antioxidants, and for their beneficial effects (antioxidant, anticancer, anti-inflammatory, antidiabetic, etc.) on human health [32]. 

Substantially, subterranean clover and sulla share proven high agronomic value and remarkable productive and qualitative traits, both representing invaluable pasture and forage resources within the communities of Mediterranean annual self-reseeding and perennial legume species. Furthermore, both legume species are currently subjected to a renewed interest not only for traditional utilization but also for their wide range of possible functions. However, despite the abundant and worldwide literature dealing with their peculiar forage traits, multiple functions, and ecosystems services, very little information is still available regarding their secondary plant metabolites, particularly the individual phenolic compounds contained in shoots. Additionally, important seasonal variations are expected according to the different growth stages of both species, which are driven by the typical conditions of Mediterranean type climate.

A study was performed in Sardinia (Italy) to evaluate the role and performance of autochthonous germplasm from native legume-based swards. In this framework, our study focused on the investigation of promising accessions, one of subterranean clover and one of sulla, as sources of phenolic compounds and antioxidants, for supporting their exploitation in the fodder, animal welfare, and nutraceutical sectors. Therefore, our specific aims were to determine the shoot content of phenolic compounds and the antioxidant capacity of both investigated leguminous species, as well as to investigate variations across the main legume growth stages.

## 2. Results and Discussion

### 2.1. Antioxidant Capacity and Phenolic Content

Phenolic compounds are major plant secondary metabolites having different chemical structure; they are necessary for a variety of plant functions and useful for many practical applications [33].

In subterranean clover, the antioxidant capacity and the content of total phenolics (TotP), non-tannic phenolics (NTP), tannic phenolics (TP), and total flavonoids (TotF) were affected significantly by legume growth stage across the season (Table 1). The results showed the highest accumulation of total phenolics at the vegetative stage (122.5 g gallic acid equivalent (GAE)·kg^−1^ DM) with considerable decrements at the flowering (61.3 g GAE·kg^−1^ DM) and seed ripening (47.3 g GAE·kg^−1^ DM) stages. The highest values of antioxidant capacity ABTS ((2,2′-azinobis (3-ethylbenzothiazoline-6-sulfonic acid) diammonium salt)) were recorded at the flower bud stage (13.6 mmol Trolox equivalent antioxidant capacity (TEAC)·100 g^−1^ DM). It is worth noting that condensed tannins (CT) were not detected at any stage. The highest TotF values were exhibited at the vegetative and flower bud stages, whereas a marked seasonal decrease occurred at the seed ripening stage. A Fertiprado commercial legume mixture (60% represented by subterranean clover) was investigated in a Mediterranean agroforestry system; in forage samples harvested in late spring, TotP contents of 62 and 67 g GAE·kg^−1^ DM were detected under partial shade below cork oaks trees and at full sunlight exposure, respectively [34], similar to our results at the flowering stage. The same authors reported a TotP content of 32 g GAE·kg^−1^ DM (full sun exposition) and 36 g GAE·kg^−1^ DM (partial shade) for *T. spumosum*, lower than our results.

For two natural populations of subterranean clover grown in Lodi (Italy) and originally collected in Sardinia, contents of 79.64 and 57.84 mg·g^−1^ DM of TotP in the leaves were reported at flowering [35], in line with our results at the same stage. They also detected very low levels of condensed tannins in leaves, whereas, in our experiment, CTs were not detected at any stage, presumably because our sample was represented by the whole shoot, which includes all the aerial plant organs.

In sulla, the antioxidant capacity and the contents of TotP, NTP, TotF, and condensed tannins (CTs) were affected significantly by plant growth stage across the season (Table 2). The peak values of antioxidant capacity, TotP, and TotF were recorded at the vegetative stage, while values were almost halved at the seed ripening stage. Both ABTS and DPPH (1,1-diphenyl-2-picrylhydrazyl) assays gave almost coincident results, and total antioxidant capacity showed a decreasing trend from the vegetative to the seed ripening stage, when values (approximately 10 mmol·100 g−^1^ DM) were half the initial ones. The levels of TotP and TotF are in line with the previous literature [36]. Burlando et al. [36] reported a content of total phenolic compounds of 68 g GAE·kg^−1^ DM at the flowering stage on aerial portions of sulla grown at two Italian sites, similar to our results; the reported content of total flavonoids at the vegetative phenological stage was also similar to our results. It is worth noting that the seasonal trend of CTs showed a peak value (25.1 g DE·kg^−1^ DM) at the flower bud stage and then a progressive reduction up to the seed ripening stage (9.4 g DE·kg^−1^ DM). Our results agree with those of Molle et al. [37] for the flower bud, while our values are slightly lower for seed ripening. Presumably, such a difference could be explained by differences in sulla genotype. In fact, we investigated a Sardinian population, whereas Molle et al. [37] used the Italian commercial variety Grimaldi. A study performed in New Zealand evidenced an extractable CT value of 4.2% *w*/*w* DM in the whole shoot of sulla in spring [38], higher than that in our results; however, the extraction method and the standard used for the quantification were different, as were the environmental conditions.

Comparatively, the total phenolic contents at the vegetative and flower bud phenological stages were approximately 30% higher in subterranean clover than in sulla. However, it is worth noting that, even with higher or comparable contents of total phenolics, subterranean clover exhibited antioxidant capacity values twofold than sulla at each investigated stage. The better performance of sulla might be explained by the distinctive presence of CTs in its shoots.

A proper content of CTs (<50 g·kg^−1^ DM) enhances forage nutritive value in grazing ruminants by reducing ruminal protein degradation and by increasing amino-acid availability in the small intestine of ruminants, without depressing rumen fiber digestion or voluntary intake. Importantly, CTs make forages bloat-safe, have positive effects on animal disease and parasite infection, and increase animal production, such as in milk and meat [30,31]. 

At the vegetative and flower bud stages, NTP values of subterranean clover were approximately twofold those of sulla (Table 1 and Table 2). The TotF content in the two legumes under study was similar only at the first two phenological stages. According to the literature, seasonal changes in the phenolic content of plants might depend on species, variety, site conditions (altitude and solar radiation), phenological stage, photoperiod, position of leaves, and abiotic and biotic stresses [34,39,40]. With regard to the phenolic content within different legume species, wide variations have been reported for the listed species from the literature (Table 3). In the subterranean clover and sulla under investigation, TotP reached peak values, whereas TotF values were similar to or lower than in the listed legumes.

It is well known that phenolic compounds contribute to the antioxidant activity in biological systems [49,50]. To estimate the inhibitory capacity of these compounds against ROS (reactive oxygen species), correlation analysis was used to investigate the relationship between phenolic content and antioxidant capacity.

For both investigated legumes, significant correlations were found between the antioxidant capacity by means of ABTS and DPPH methods and the phenolic contents. Subterranean clover showed a highly significant correlation between antioxidant capacity and total phenolics (*R^2^* = 0.9470) (Table 4). Significant correlations were also established between DPPH and TotF contents (*R^2^* = 0.9520). Our results agree with other studies that reported a relationship between antioxidant capacity and total phenolic compound content [34,51,52,53].

In sulla, ABTS and DPPH showed a highly significant correlation with total phenolics (*R^2^* = 0.9372 and 0.9326, respectively; Table 5). Significant correlations were also established between antioxidant capacity (ABTS and DPPH) and TP content, reaching significant correlations of *R^2^* = 0.9282 and 0.9053, respectively. These correlations indicate that the level of antioxidant activity could be attributed to the presence of antioxidant phenolic compounds, expressed in different quantities, according to their physiological or ecological function at each different phenological stage. As expected, a significant positive correlation *(p* ≤ 0.01) was found between CT and antioxidant capacity.

### 2.2. RP-HPLC Analysis of Phenolic Compounds

Phenolic compounds are the most studied group of plant-specialized metabolites, which include more than 8000 molecules. They are biosynthesized through a shikimate/phenylpropanoid pathway that produces a wide array of monomeric and polymeric polyphenols. The chemical structure of phenolic compounds might vary; however, a common trait is the presence of one or more hydroxyl substituents, attached directly to one or more aromatic or benzene rings. On the basis of their structure, they may be grouped into phenolic acids, flavonoids, stilbenoids, and lignans. Usually, phenolic compounds are present as free form in plants; however, more often, they are found with one or more sugars residues linked by β-glicosidic bonds to a hydroxyl group (*O*-glycosides) or a carbon atom of the aromatic ring (*C*-glycosides) [39].

Even though phenolic compounds are often considered as a group of molecules with similar biological activity, their different chemical structures significantly influence their activity and role in biological processes [39].

A total of 10 individual phenolic compounds were detected in the shoot of subterranean clover; among them, 7 were identified at 4 different growth stages (Table 6). Three hydroxycinnamic acid derivatives (chlorogenic and cryptochlorogenic acids, as well as a hydroxycinnamic acid derivative, quantified as caffeic acid equivalent), the flavanol quercetin 3-glucoside, and the flavone luteolin were detected. 

The isoflavones genistin (genistein 7-glucoside), biochanin A (5,7-dihydroxy-4′-methoxyisoflavone), and sissotrin (biochanin A 7-*O*-β-d-glucopyranoside) were also identified in subterranean clover extracts. In addition, on the basis of absorption spectra identical to the isoflavonones genistin, sissotrin, and biochanin A, two peaks with the maximum absorption at 280 nm were indicated as isoflavone derivative form I and isoflavone derivative form II, quantified as biochanin A equivalents. 

Concerning quantitative analysis, the highest values of the individual phenolic sum were recorded at the vegetative and flower bud stages, with decreases of 46% and 22% at the flowering and seed ripening stages, compared to the vegetative stage. Isoflavones were the most abundant molecules across all phenological stages of subterranean clover; in particular, derivative isoflavones (I and II) showed the highest concentrations, reaching values of 18.27 and 15.29 mg·g^−1^ DM at the flowering and vegetative stages, respectively.

Tava et al. [54] reported phenolic quantitative analysis in leaves of 14 genotypes of subterranean clover, sampled at the plant stage of full bloom/beginning of burr formation. The chromatographic profile shown by Tava et al. [54] is very similar to our results; therefore, we might suppose that isoflavone I coincides with genistein-7-*O*-β-d-glucoside-6″-*O*-malonate, and isoflavone II corresponds to biochanin A-7-O-β-d-glucoside-6″-*O*-malonate. Furthermore, these two molecules had the highest concentration among all accessions of subterranean clover studied by Tava et al. [54], in agreement with our results. The same authors observed a great variation in isoflavone content within subterranean clover genotypes; however, our values of genistin, sissotrin, and biochanin A are inside the range reported by them. Again, the content of quercetin-3-*O*-β-d-glucoside (isoquercitrin) was very close to our data at the flowering stage. Formononetin and ononin (formononetin 7-*O*-glucoside) were also detected in genotypes of subterranean clover by Tava et al. [54,55]. It is worth noting that these compounds were not detected in our study. Considerable amounts of formononetin and its highly estrogenic rumen metabolite equol are well known for their detrimental effects on fertility and reproduction in ruminants [56]. Therefore, the absence of these compounds in our study represents a valuable and distinctive feature of our investigated subterranean clover genotype.

In a study performed in Australia on *T. subterranean* subsp. *yanninicum* ecotypes derived from wild Mediterranean populations, leaf isoflavone content (formononetin, genistein, and biochanin A) was investigated only at the flowering stage using thin-layer chromatography [57]. A study on five different clover species, namely, *T. arvense*, *T. campestre*, *T. dubium*, *T. hybridum*, and *T medium*, grown and harvested in Bulgaria at the full bloom stage, reported the presence of biochanin A (0.174–0.503 mg·g^−1^ DM), genistein, daidzein, and formononetin, differing according to the field slope and species [58]. A biochanin A content of 0.503 mg·g^−1^ DM was detected in *T. hybridum*, close to our results at the flowering stage in subterranean clover.

The individual phenolic content in sulla shoots at different growth stages is shown in Table 7. A total of 11 individual phenolic compounds were identified in sulla shoots:

- Three isomers of caffeoylquinic acid, i.e., neochlorogenic, chlorogenic, and cryptochlorogenic acids;
- Caffeic acid and a hydroxycinnamic acid derivative (absorption spectrum identical to caffeic acid, quantified as caffeic acid equivalent);
- Five flavanols, i.e., rutin, quercetin 3-galattoside, quercetin 3-glucoside, isorhamnetin 3-rutinoside, and one of its isomers (quantified as isorhamnetin 3-rutinoside equivalent);
- Diosmin, a flavone glycoside of diosmetin.

Concerning quantitative analysis, the highest values of the individual phenolics were recorded at the vegetative stage, similarly to TotP trend observed with the Folin–Ciocalteau assay. A decreasing trend in the individual phenolic sum by 35%, 48%, and 66% was observed at the flower bud, flowering, and seed ripening stages compared to the vegetative stage. 

Statistical analysis evidenced variations according to phenological stage for all individual phenolic compounds except for quercetin 3-glucoside. Chlorogenic acid (0.76–3.43 mg·g^−1^ DM) and diosmin (3.64–4.94 mg·g^−1^ DM) were the most abundant compounds at the vegetative and flower bud stages.

Tava et al. [55] reported the flavonoid content in the methanolic purified extracts of sulla flowers and leaves. Similar to our data, they detected quercetin and isorhamnetin, along with their isomers or derivatives, as the dominant aglycones forming the different flavonoids, but they did not detect any phenolic acid or diosmin. Furthermore, they found formononetin and afrormosin isoflavones, which were not detected in our samples. Conversely, Burlando et al. [36] identified diosmin in aerial parts of two accessions of sulla. Another study performed with mass spectrometric analysis identified chlorogenic acid, quercetin-3-*O*-rutinoside (rutin), and quercetin 3-d-galattoside (hyperoside) in sulla grown in New Zealand [38], in line with our results. The same authors found kaempferol 3-*O*-rutinoside, but this flavanol was not detected in our study, with the same observation for ononin (formononetin 7-*O*-β-glucopyranoside). A review reported that chlorogenic acid and several glycosyl derivatives of the flavones quercetin and kaempferol and the glycosyl malonate derivatives of the isoflavones genistein and formononetin have been detected in the aerial parts of sulla [41]. 

Neochlorogenic acid was evidenced at the seed ripening stage (0.10 mg·g^−1^ DM), whereas it was found in trace amounts at other phenological stages. Chlorogenic acid and diosmin were the most abundant compounds at the vegetative (3.36 and 4.94 mg·g^−1^ DM, respectively) and flower bud (3.43 and 3.76 mg·g^−1^ DM) stages. At the seed ripening stage, the highest content was shown by diosmin with a value of 3.64 mg·g^−1^ DM. 

Caffeic acid showed the highest content at the seed ripening stage, with threefold higher values than that at vegetative and flower bud stages. According to Riaz et al. [59], caffeic acid is involved in the regulation of cell expansion, turgor pressure, and lignin synthesis.

Isorhamnetin 3-*O*-rutinoside and its isomer were detected at all phenological stages (0.27–1.23 mg·g^−1^ DM and 0.03–0.29 mg·g^−1^ DM), both reaching their maximum concentration at the flower bud stage, in accordance with Tava et al. [55], who detected similar values in the leaves and flowers. The same authors [55] also reported a higher concentration of rutin (quercetin 3-*O* rutinoside) in sulla leaves (0.71 mg·g^−1^ DM) than that in our data; however, a direct comparison with the literature is possible only for extractions from the whole shoot. Future investigations are required to examine the individual phenolic compounds in different parts of the plant (leaflets, petioles, stems, and racemes) at different phenological stages.

Chlorogenic acid is the most abundant among the caffeoyl quinic acid isomers in nature [60]. It is widely recognized to have antioxidant activities and a wide range of differing biological effects; it has been proven to be an efficient defense molecule against a broad range of insect herbivores in different plant species.

To our knowledge, this is the first study regarding the antioxidant capacity and individual phenolic compounds quantification in sulla and subterranean clover shoots, from the vegetative to the seed ripening stages. Additionally, our study indicated that genistin, biochanin A and its derivatives, sissotrin, and luteolin were exclusively present in subterranean clover, whereas isorhamnetin 3-rutinoside, its isomer diosmin, and rutin were present only in sulla. 

Forage plants represent a rich and interesting source of bioactive compounds that are specific to each forage species [41]. However, when considering the common individual phenolic compounds, namely, chlorogenic and cryptochlorogenic acids, hydroxycinnamic acid derivatives, and quercetin 3-glucoside (Table 6 and Table 7), our findings indicate that their contents widely differed according to species and growth stages. 

**Figure 1:**
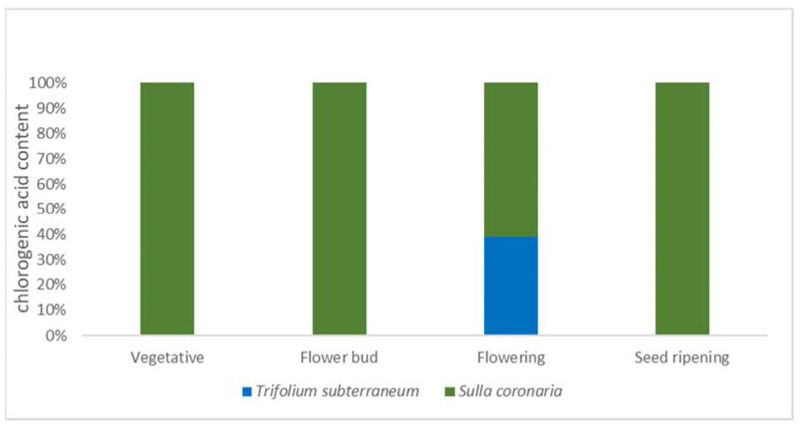
Cumulated content of chlorogenic acid from *Trifolium subterraneum* and *Sulla coronaria* shoots across phenological stages.

Therefore, our results suggest considering a combined utilization of both legume species according to space (e.g., different fields and/or farms) and time. This might ensure a prolonged supply across all phenological stages of a given common bioactive compound with related potential benefits for animal production and health (e.g., chlorogenic acid; see Figure 1). Furthermore, the peculiar composition in bioactive compounds of the abovementioned species must be considered as an important feature in addition to the traditional productive and qualitative traits usually considered.

Regression analysis was used to explore the relationships between individual phenolic compounds and the antioxidant capacity, total phenolics, and condensed tannins; in subterranean clover, seven individual phenolic compounds were highly correlated with antioxidant capacity, TotP, NTP, and TotF (Table 8), except for sissotrin, which presented a moderate correlation (*p* ≤ 0.05).

In sulla, a high positive correlation was observed among all the studied variables and chlorogenic acid and quercetin 3-galattoside, whereas diosmin and hydroxycinnamic acid derivatives were highly correlated with NTP (Table 9).

A high correlation between antioxidant capacity and chlorogenic acid was found in plum fruit [61], whereas quercetin 3-galactoside was found highly correlated to total antioxidant activity in the flesh of ‘Cripps Pink’ apples [62]. Isorhamnetin derivatives presented a moderate or nonsignificant correlation with the studied variables except for CTs (*R^2^* = 0.8001 and 0.8675, respectively). Our study revealed that phenolics, particularly flavonoids, may be the main contributors to the antioxidant activity exhibited by plants, in agreement with Burlando et al. [36], who outlined the well-known capacity of flavonoids in determining the biological properties of herbal products. Therefore, a higher content of these compounds in a phytocomplex is predictive of stronger bioactivity. Tava et al. [41] reported that *Trifolium* spp. produce a range of specialized metabolites. Among phenolics, flavonoids (particularly isoflavones) are the most investigated as natural growth-promoting agents and for their effects in animal nutrition.

Lastly, we totally agree with Tava et al. [41], who stated that forage legume species represent an interestingly rich source of bioactive compounds for the presence of a great variety of molecular structures to be employed in the pharmaceutical and agro-industry sectors.

## 3. Materials and Methods 

The field experiments were carried out in Sardinia (Italy), where the climate is Mediterranean with a mild winter. Subterranean clover and sulla shoot samples were collected from two distinctive sites of Sardinia. The former location (central Sardinia, 40° N, 8° E, 200 m a.s.l.) has a subacid, sandy clay loam (*Eutric, Mollic Fluvisols*), with a long-term average annual rainfall of 580 mm and a mean annual air temperature of 16.6 °C. The second location (north Sardinia, 41° N, 8° E, 81 m a.s.l.) has a subalkaline, sandy clay loam soil overlaid on limestone (*Xerochrepts*), with a long-term average annual rainfall of 554 mm and a mean annual air temperature of 16.2 °C.

### 3.1. Plant Material and Shoot Sampling

**Figure 2:**
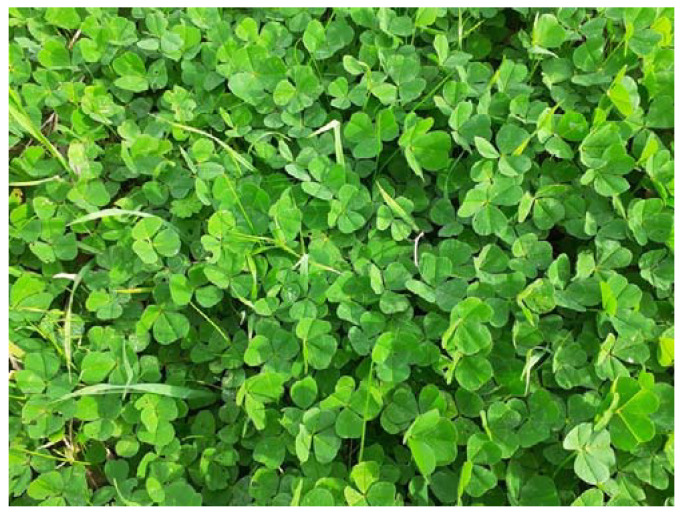
*Trifolium subterranean* TS007 at vegetative stage.

**Figure 3:**
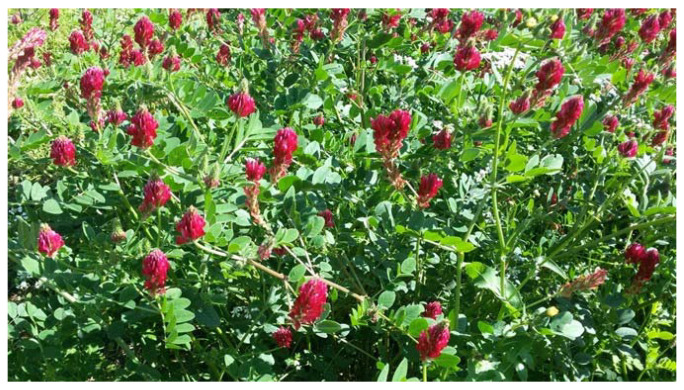
*Sulla coronaria* SC004 at flowering stage.

A native population of subterranean clover (TS007) at the first site and a native population of sulla (SC004) at the second site (Figure 2 and Figure 3) were monitored, and their shoots were repeatedly sampled from January to June 2020, according to different plant growth stages, namely, the vegetative, flower bud, flowering, and seed ripening stages.

Samples of the two forages were immediately frozen and stored at −20 °C, before freeze-drying using a Heto Lyolab 3000 (Heto-Holten A/S, Allerød, Denmark) for 48 h (−55 °C). After lyophilization, the samples were ground in a mill to a fine powder and stored in total darkness at −20 °C until further analyses. Sample preparation procedures were performed according to Molinu et al. [63]. Briefly, 50 mg of the lyophilized sample was extracted with a 2.5 mL methanol/water (80:20 *v*/*v*) mixture and shaken for 24 h. Homogenates were centrifuged (10 min at 3900 rpm), and the organic extracts were filtered using 0.20 µm polytetrafluoroethylene syringe filters, before being stored at −20 °C until analysis. Methanolic extracts were then analyzed for antioxidant activity, total phenolics, non-tannic phenolics, tannic phenolics, condensed tannins, and individual phenolic compounds. All samples were analyzed in triplicate.

### 3.2. Antioxidant Capacity and Total Phenolic Content 

Antioxidant capacity was evaluated by means of the ABTS ((2,2′-azinobis (3-ethylbenzothiazoline-6-sulfonic acid) diammonium salt)) and DPPH (1,1-diphenyl-2-picrylhydrazyl) assays, as reported by Sanna et al. [64]. The results are expressed in terms of Trolox equivalent antioxidant capacity (TEAC), as mmol Trolox equivalents·100 g^−1^ dry matter of shoots (mmol TEAC·100 g^−1^ DM).

Total phenolics (TotP), non-tannic phenolics (NTP), and tannic phenolics (TP) of extracts were evaluated using spectrophotometric analysis with Folin–Ciocalteau reagent, according to procedures previously described [64]. The results are expressed as g of gallic acid equivalent (GAE)·kg^−1^ dry matter of plant material (g GAE·kg^−1^ DM) by means of a calibration curve of gallic acid. Total flavonoids (TotF) were quantified by colorimetric assay using the AlCl_3_ method, following procedures previously reported [64]. TotF was quantified using a catechin calibration curve, and the results are expressed as g of catechin equivalent (CE)·kg^−1^ dry matter (g CE·kg^−1^ DM). 

The butanol assay was used for quantification of the extractable condensed tannin content from the sample, expressed as g delphinidin equivalent (DE)·kg^−1^ DM [34].

### 3.3. RP-HPLC Analysis of Phenolic Compounds

Individual phenolic compounds were detected in methanolic extracts, which were prepared as previously described (Section 3.1).

Chromatographic separation was carried out according to Molinu et al. [63] via an RP-HPLC method using an Agilent 1260 series HPLC instrument (Agilent Technologies, Palo Alto, CA, USA) equipped with a quaternary pump (G1311B), degasser, column thermostat (G1316A), auto-sampler (G1329B), and diode array detector (G1315B, DAD). Briefly, the column was a Zorbax Eclipse plus C18 (250 × 4.6 mm, 5 µm; Agilent); the column temperature was set to 30 °C and the flow rate was 0.8 mL·min^−1^. The injection volume was 10 μL, and the detection wavelengths were set to 280, 330, and 350 nm. Elution was carried out with a binary mobile phase of solvent A (water and 0.1% trifluoroacetic acid) and solvent B (acetonitrile). Data were processed using the Agilent OpenLAB CDS ChemStation edition 2012. Molecule identifications were achieved as a function of the retention time of available standards, which were selected from the literature concerning their phenolic composition in sulla and *Trifolium* spp. [36,38,41,54,55,58] and their UV absorption spectra, as well as by adding standard solutions to the sample. Quantification of individual phenolic compounds was performed using the external standard method curve (five known concentrations for each standard in duplicate; *R^2^* = 0.99) and expressed in mg·g^−1^ DM.

### 3.4. Data Analyses

Results of chemical determinations carried out on shoot samples were subjected to a one-way analysis of variance, using Statgraphics Centurion XVI version [65] (StatPoint Technologies Inc. 2009), to test the effect of different phenological stages on the following variables: concentrations for antioxidant capacity, total phenolics, total flavonoids, and individual phenolic compounds. Differences between means were assessed using Fisher’s least significant difference (LSD) procedure for means separation. The significance level was fixed at *p* ≤ 0.05 for all the statistical analyses. The regression analyses between polyphenols and antioxidant capacity were calculated using Microsoft Excel, Microsoft 365 MSO.

## 4. Conclusions

The present study is the first report of the antioxidant activity and quantification of individual phenolic compounds from the aerial parts of subterranean clover and sulla populations grown in a Mediterranean environment and harvested at different phenological stages.

In both legume species under study, qualitative and quantitative differences in chemical composition were detected across different growth stages. Our research evidenced that the antioxidant capacity and the content of total phenolic compounds were affected by growth stage, with the highest contents at the vegetative stage. Isoflavones in subterranean clover and chlorogenic acid and diosmin in sulla were the main bioactive compounds isolated from their shoots.

Our results suggest a combined utilization of both legume species in space and time to ensure a prolonged supply of a given bioactive compound across all phenological stages. Furthermore, the peculiar composition of phenolic compounds in the abovementioned species must be considered as an important feature, in addition to the traditional productive and qualitative traits, in view of the potential benefits for animal production and health.

## Figures and Tables

**Table 1:** Total antioxidant capacity (TEAC) by ABTS and DPPH methods, total phenolics (TotP), non-tannic phenolics (NTP), tannic phenolics (TP), and total flavonoids (TotF) in the examined *Trifolium subterraneum* shoots.

Phenological Stage	TEAC (mmol 100 g^−1^ DM)		TotP (g GAE kg^−1^ DM)	NTP (g GAE kg^−1^ DM)	TP (g GAE kg^−1^ DM)	TotF (g CE kg^−1^ DM)
	ABTS	DPPH				
Vegetative	11.6 b	10.6 a	122.5 a	64.2 a	58.3 a	10.1 a
Flower bud	13.6 a	9.2 b	108.5 b	57.7 a	50.8 a	9.5 b
Flowering	7.4 c	5.2 c	61.3 c	34.1 b	27.2 b	5.2 c
Seed ripening	6.2 d	4.4 d	47.3 d	23.9 c	23.4 b	3.4 d

In the columns means followed by the same letter are not significantly different at *p* ≤ 0.05.

**Table 2:** Total antioxidant capacity (TEAC) by ABTS and DPPH methods, total phenolics (TotP), non-tannic phenolics (NTP), tannic phenolics (TP), and total flavonoids (TotF) and extractable condensed tannins (CT) in the examined *Sulla coronaria* shoots.

Phenological Stage	TEAC (mmol 100 g^−1^ DM)		TotP (g GAE kg^−1^ DM)	NTP (g GAE kg^−1^ DM)	TP (g GAE kg^−1^ DM)	TotF (g CE kg^−1^ DM)	CT (g DE kg^−1^ DM)
	ABTS	DPPH					
Vegetative	20.3 a	20.4 a	92.7 a	28.4 a	64.3 a	11.4 a	20.3 b
Flower bud	18.4 b	18.0 b	81.3 b	22.6 b	58.7 a	10.0 b	25.1 a
Flowering	14.4 c	14.6 c	71.6 c	22.3 b	49.2 b	9.1 c	16.6 c
Seed ripening	10.1 d	10.0 d	54.3 d	20.2 c	34.1 c	5.3 d	9.4 d

In the columns, means followed by the same letter are not significantly different at *p* ≤ 0.05.

**Table 3:** Total phenolics (TotP), non-tannic phenolics (NTP), tannic phenolics (TP), and total flavonoids (TotF) in different legume species (mg g^−1^ DM).

		TotP	NTP	TP	TotF	References
Scientific Name	Common Name					
*Trifolium subterraneum*	Subterranean clover	47–122	24–64	23–58	3–10	Authors
*Sulla coronaria*	Sulla	54–93	20–28	34–64	5–11	Authors
*Trifolium spumosum*	Bladder clover	32–36	20–32	15–16	2–3	[34]
*Trifolium alessandrinum*	Berseem clover	27–62	n.a.	n.a.	n.a.	[41]
*Trifolium pratense*	Red clover	8–73	n.a.	n.a.	n.a.	[41]
*Trifolium repens*	White clover	8–16	n.a.	n.a.	n.a.	[41]
*Medicago sativa*	Lucerne	12–16	n.a.	n.a.	n.a.	[42]
*Medicago minima*	Bur medick	17	n.a.	n.a.	10	[43]
*Hedysarum aucheri*	Unknown	16–122	n.a.	n.a.	9–66	[44]
*Hedysarum cappadocicum*	Tatli tirfil	51	n.a.	n.a.	n.a.	[45]
*Onobrychis nitida*	Firat korungasi	102	n.a.	n.a.	n.a.	[45]
*Onobrychis viciifolia*	Sainfoin	40–57	15–17	n.a.	n.a.	[46]
*Bituminaria bituminosa*	Tedera	13	n.a.	n.a.	n.a.	[47]
*Chamaecytisus proliferus*	Tagasaste	33–112	40–96	3–20	20–68	[47,48]

n.a. = not available.

**Table 4:** Correlations (*R^2^*) established between total phenolics (TotP), non-tannic phenolics (NTP), tannic phenolics (TP), total flavonoids (TotF) and antioxidant capacity (ABTS, DPPH) in *Trifolium subterraneum* shoots.

Subterranean Clover			
	ABTS	DPPH	TotP
DPPH	0.7866 ***		
TotP	0.8226 ***	0.9470 ***	
NTP	0.7656 ***	0.9242 **	0.9433 ***
TP	0.7786 ***	0.8506 ***	0.9328 ***
TotF	0.8744 ***	0.9520 ***	0.9800 ***

*** *p* ≤ 0.001, ** *p* ≤ 0.01.

**Table 5:** Correlations (*R^2^*) established between total phenolics (TotP), non-tannic phenolics (NTP), tannic phenolics (TP), total flavonoids (TotF), condensed tannins and antioxidant capacity (ABTS, DPPH) in *Sulla coronaria* shoots.

Sulla			
	ABTS	DPPH	TotP
DPPH	0.9448 ***		
TotP	0.9372 ***	0.9326 ***	
NTP	0.6776 **	0.7343 **	0.7496 **
TP	0.9282 ***	0.9053 ***	0.9821 ***
TotF	0.9116 ***	0.9110 ***	0.9466 ***
CT	0.7630 **	0.6660 **	0.6398 *

*** *p* ≤ 0.001, ** *p* ≤ 0.01, * *p* ≤ 0.05.

**Table 6:** HPLC analysis of individual phenolic compounds (mg g^−1^ DM) in *Trifolium subterraneum* shoots.

Phenolic Compounds	*t_R_ (min)	**λmax (nm)	Vegetative	Flower Bud	Flowering	Seed Ripening
Chlorogenic acid	10.69	330	nd	nd	1.22	nd
Cryptochlorogenic acid	11.27	330	nd	nd	tr	nd
Hydroxycinnamic acid derivative	18.10	330	3.70 b	4.90 a	0.45 c	0.44 c
Quercetin 3-glucoside	21.90	350	3.49 a	2.78 b	1.66 c	0.87 d
Genistin	22.65	280	7.66 b	9.38 a	4.46 c	2.13 d
Isoflavone I	28.04	280	17.38 a	18.27 a	7.22 b	3.60 c
Luteolin	34.05	350	0.43 a	0.27 b	tr	nd
Sissotrin	35.16	280	1.01 a	0.97 a	0.97 a	0.45 b
Isoflavone II	37.36	280	15.29 a	12.62 b	6.37 c	3.19 d
Formononetin	38.60	280	nd	nd	nd	nd
Biochanin A	41.70	280	0.85 a	0.72 b	0.55 c	0.41 d
Sum			49.81	49.91	22.90	11.09

*t_R_ = retention time; **λmax = wavelengths of maximum absorption in the UV region. In the rows, means followed by the same letter are not significantly different at *p* ≤ 0.05. nd = not detected; tr = trace.

**Table 7:** HPLC analysis of individual phenolic compounds (mg g^−1^ DM) in *Sulla coronaria* shoots.

Phenolic Compounds	*t_R_ (min)	**λmax (nm)	Vegetative	Flower Bud	Flowering	Seed Ripening
Neochlorogenic acid	9.45	330	tr	tr	tr	0.10
Chlorogenic acid	10.69	330	3.36 a	3.43 a	1.91 b	0.76 c
Cryptochlorogenic acid	11.27	330	0.56 a	0.28 b	0.25 b	0.25 b
Caffeic acid	14.33	330	0.10 b	0.09 b	0.03 c	0.30 a
Hydroxycinnamic acid derivative	15.90	330	2.47 a	0.33 c	0.84 b	0.35 c
Rutin	19.91	350	0.38 a	0.17 b	0.18 b	0.05 c
Quercetin 3-galattoside	20.10	350	1.00 a	0.67 b	0.67 b	0.19 c
Quercetin 3-glucoside	21.00	350	0.11 a	0.12 a	0.12 a	0.11 a
Isorhamnetin 3-rutinoside	23.19	350	0.64 b	1.23 a	0.43 c	0.27 d
Isorhamnetin 3-rutinoside (isomer)	23.52	350	0.17 b	0.29 a	0.09 c	0.03 d
Diosmin	24.06	350	4.94 a	3.76 bc	3.93 b	3.64 c
Sum			13.73	10.37	8.45	6.05

*t_R_ = retention time; **λmax = wavelengths of maximum absorption in the UV region. In the rows, means followed by the same letter are not significantly different at *p* ≤ 0.05. nd = not detected; tr = trace.

**Table 8:** Correlations (*R^2^*) established between antioxidant capacity (ABTS, DPPH), total phenolics (TotP), non-tannic phenolics (NTP), tannic phenolics (TP), total flavonoids (TotF), and individual phenolic compounds in *Trifolium subterraneum* shoots.

	ABTS	DPPH	TotP	NTP	TP	TotF
Hydroxycinnamic acid derivative	0.9700 ***	0.8071 ***	0.8293 ***	0.7614 **	0.7966 ***	0.8624 ***
Quercetin 3-glucoside	0.7486 **	0.9136 ***	0.9612 ***	0.9028 ***	0.9007 ***	0.9546 ***
Genistin	0.9653 ***	0.7808 ***	0.8367 ***	0.8120 ***	0.7571 **	0.9003 ***
Isoflavone I	0.9459 ***	0.8929 ***	0.9479 ***	0.8924 ***	0.8861 ***	0.9749 ***
Luteolin	0.7154 **	0.9436 ***	0.9462 ***	0.8719 ***	0.9052 ***	0.9002 ***
Sissotrin	0.4400 *	0.4232 *	0.4654 *	0.4621 *	0.4098 *	0.5211 *
Isoflavone II	0.8024 ***	0.9271 ***	0.9815 ***	0.9119 ***	0.9306 ***	0.9712 ***
Biochanin A	0.7356 **	0.9156 ***	0.9543 ***	0.9021 ***	0.8879 ***	0.9502 ***

*** *p* ≤ 0.001, ** *p* ≤ 0.01, * *p* ≤ 0.05.

**Table 9:** Correlations (*R^2^*) established between antioxidant capacity (ABTS, DPPH), total phenolics (TotP), non-tannic phenolics (NTP), tannic phenolics (TP), total flavonoids (TotF), condensed tannins (CT) and individual phenolic compounds in *Sulla coronaria* shoots.

	ABTS	DPPH	TotP	NTP	TP	TotF	CT
Chlorogenic acid	0.9489 ***	0.9136 ***	0.8544 ***	0.5105 *	0.8817 ***	0.8404 ***	0.8697 ***
Cryptochlorogenic acid	0.4997 *	0.5316 *	0.5605 *	0.9027 ***	0.4371 *	0.4305 *	0.0920 ns
Caffeic acid	0.4200 *	0.4373 *	0.4486 *	0.1865 ns	0.4947 *	0.6311 *	0.4716 *
Hydroxycinnamic acid derivative	0.4023 *	0.4400 *	0.5082 *	0.8649 ***	0.3879 *	0.4272 *	0.0450 ns
Rutin	0.7321 **	0.7754 **	0.8148 ***	0.9413 ***	0.7125 **	0.7677 **	0.2869 ns
Quercetin 3-galattoside	0.8459 ***	0.8600 ***	0.8988 ***	0.7645 ***	0.8561 ***	0.9177 ***	0.5207 *
Quercetin 3-glucoside	0.0031 ns	0.0022 ns	0.0020 ns	0.0882 ns	0.0180 ns	0.0059 ns	0.1348 ns
Isorhamnetin 3-rutinoside	0.4669 *	0.4186 *	0.3276 *	0.0495 ns	0.4099 *	0.3341 *	0.8001 ***
Isorhamnetin 3-rutinoside isomer	0.6073 *	0.5625 *	0.4746 *	0.1311 ns	0.5557 *	0.4845 *	0.8675 ***
Diosmin	0.4726 *	0.5165 *	0.5698 *	0.8652 ***	0.4513 *	0.5052 *	0.0990 ns

ns = not significant; *** *p* ≤ 0.001, ** *p* ≤ 0. 01, * *p* ≤ 0.05.

## Data Availability

Not applicable.
